# Eco-friendly inhibitors for sustainable corrosion protection of mild steel under oilfield acidizing conditions

**DOI:** 10.1038/s41598-025-27271-8

**Published:** 2025-11-27

**Authors:** E. Khamis, A. M. Abdel-Gaber, M. Morshidy, M. E. Mohamed

**Affiliations:** 1 Chemistry Department, Faculty of Science, Alexandria University, Alexandria, Egypt; 2 Science & Innovation Center of Excellence, SICE, Egyptian Russian University, Badr, Egypt; 3 Arab Pipelines Petroleum Company (SUMED), Sidi Kerir Terminal, Alexandria, Egypt

**Keywords:** Mild steel, Acid corrosion, Oil well acidizing, Green corrosion inhibitors, Temperature effect, Chemistry, Engineering, Materials science

## Abstract

This study investigates the effect of temperature (30–60 °C) on the corrosion behavior of mild steel in 15% hydrochloric acid, in the absence and presence of four novel, eco-friendly corrosion inhibitors derived from ethanolic extracts of agro-industrial and botanical wastes. Electrochemical impedance spectroscopy (EIS), potentiodynamic polarization, adsorption isotherm, and activation parameter determination were employed, alongside surface morphology analysis using scanning electron microscopy (SEM). All extracts acted as mixed-type inhibitors, achieving high efficiency at low concentrations (0.010–0.600 g/L), with inhibition rates decreasing slightly from 85.8 to 78.4% at 30 °C to 81.9–61.8% at 60 °C. SEM confirmed the formation of a protective surface film, while adsorption followed Langmuir, Flory–Huggins, and Temkin isotherms. Thermodynamic analysis indicated a spontaneous process involving both physisorption and chemisorption. These findings highlight the thermal stability, strong adsorption capability, and practical potential of the studied plant-based extracts as sustainable, non-toxic alternatives to conventional inhibitors for use in harsh oilfield acidizing and descaling operations.

## Introduction

Oil and natural gas continue to dominate global energy supply, accounting for 60% of total consumption, making it unlikely that traditional fossil fuel extraction methods will phase out in the coming decades^1^. Acidic solutions, particularly hydrochloric acid (HCl, 5–28%), are routinely employed in the oil and gas sector for operations such as acid cleaning, well acidizing, pickling, descaling, and various petrochemical processes. While these applications are effective, they often accelerate metal dissolution and corrosion of steel components. Mild steel remains a preferred material in petroleum infrastructure, construction, and mechanical systems owing to its cost-effectiveness, affordability, strong mechanical properties, and high tensile strength. However, it is highly prone to corrosion at every stage-from extraction and refining to storage and transportation. This issue often manifests as leakage in tanks, pipelines, tubing, and casings. Although total corrosion prevention is not feasible, effective mitigation strategies can significantly reduce corrosion-related damage. Among these, corrosion inhibitors (CIs) are widely favored due to their practicality, adaptability, efficiency, and cost-effectiveness^1–4^. Even in trace concentrations, CIs can markedly reduce corrosion rates in aggressive environments, though their effectiveness varies depending on the specific metal, corrosive medium, and operating conditions^1–3^. The selection and optimal dosage of a corrosion inhibitor rely on various factors, such as the concentration and type of acid used, the nature of the metal substrate, and the maximum operating temperature^1^.

Elevated downhole temperatures significantly affect the performance of CIs during well stimulation treatments. Processes such as pickling, typically conducted at high temperatures, can degrade thermally sensitive inhibitor components, thereby diminishing their effectiveness^1,2^. Given that geothermal gradients average approximately 25 °C per kilometer depth, downhole environments frequently reach elevated temperatures that not only accelerate corrosion processes but also interfere with inhibitor adsorption mechanisms. At higher temperatures, corrosion rates increase exponentially due to the reduction in hydrogen evolution overpotential and the deterioration of protective metal surface-inhibitor films, resulting in decreased inhibition efficiency (IE)^2,5^. Temperature fluctuations also contribute critically in influencing the behavior of metal corrosion and the nature of inhibitor adsorption. Thermal effects at the metal-inhibitor interface can induce complex changes, such as rearrangement or decomposition of inhibitor molecules, intensified rapid acid-metal interactions, and desorption of inhibitors from the metal surface. Studying these temperature-dependent phenomena enables the calculation of thermodynamic corrosion parameters, and offers insights into the type and mechanism of adsorption (physical vs. chemical)^3,6,7^. In industrial practice, acid pickling is performed for steel at high temperatures— typically for hydrochloric acid (HCl) up to 60 °C, and for sulfuric acid (H_2_SO_4_) up to 90 °C. Consequently, effective corrosion inhibitors must possess excellent thermal stability to maintain high protection efficiencies under such aggressive and corrosive conditions^4,7^.

Unfortunately, many of the commonly used CIs are highly toxic, costly, and pose significant environmental and health risks, thus limiting their industrial applicability. In response to increasing environmental regulations and sustainability concerns, recent research has focused on the exploration of natural product-based alternatives. These green inhibitors are favored for their environmental compatibility, low cost, abundance, non-toxicity, renewability, and biodegradability^2,8^.

A review of the literature on steel corrosion inhibition in 15% hydrochloric acid (HCl) at elevated temperatures reveals that most studies have employed synthetic chemical inhibitors^1,2,9,10^. In contrast, relatively few studies have investigated the efficiency of plant-based extracts under such conditions^3–6^, and it is particularly rare to find research examining the use of plant extracts as corrosion inhibitors for mild steel in 15% HCl at high temperatures^11–14^.

A. Farhadian et al.^3^ studied the efficiency of castor oil in corrosion inhibition in 1 M HCl on MS using 140 µM. IE increased from 82% at 20 °C to 91% at 80 °C. S. Chen et al.^5^ used 0.3 g/L of Solanum Tuberosum leaf extract, an agricultural organic waste, as CI for Q235 steel in 1 M HCl achieving 92% at 293 K that decreased to 74% at 313 K. However, the Fig and olive by-products, an aqueous leaves extract of 1:1 mixture using 0.25 mL.mL^− 1^, tested by H. Rahmouni et al.^4^, resulted in an inhibition of 92–95% on increasing from 298 to 338 K. P. Ghahremani et al.^11^ investigated Golpar leaves extract for MS in 1 M HCl that maintained IE of 89–90% from 25 to 45 °C, while Araucaria Heterophylla leaves extract tested by V. Singh and his coworkers^12^ decreased from 84% to 62% on increasing from 298 to 328 K using 1000 ppm concentration. A. Kumar et al.^13^ examined Chayote (Sechium edule) fruit extract using 2000 ppm for MS in 1 M HCl at 303 K achieving IE of 77% that became 62% at 323 K. M.A. Deyab et al.^14^ utilized Equisetum arvense, 300 ppm aerial part extract, for carbon steel in 2 M HCl inhibiting by 95% at 298 K that deceased to 89% at 328 K, while 0.08 g/mL of Eucalyptus Camaldulensis leaves extract examined by L. Ghalib et al.^6^ under same conditions at 20 °C gave an IE of 92% that became 61% at 60 °C. I.H.N. Keivani et al.^15^ investigated Pomegranate peel extract from 1:2 (V/V) water/acetonitrile. At concentration of 2 g/L, IE decreased from 97% at 20 °C to 65% at 60 °C in 15% HCl for carbon steel. R.H. Aldahiri and his collaboration^7^ examined Canarium Strictum leaves ethanolic extract for MS in 15% HCl using 700 ppm that downed from 92 to 64% on raising temperature from 293 at 323 K. M.A. Deyab et al.^16^ investigated Lavender angustifolia extract for 316 stainless steel/5 M HCl system, resulting in an IE of 92 − 84% on raising from 298 to 33 3 K using 300 ppm extract. S.A. Umoren and his scholars^8^ tested Leaf (L) and seed (S) aqueous extracts of date palm for X60 carbon steel in 15% HCl. An extract of 2000 ppm of L decreased from 83 to 74%, while that of S decreased from 77 to 60% on raising the temperature from 25 to 60 °C.

Previous studies on plant- and agro-waste-based corrosion inhibitors in acidic media consistently demonstrate that inhibition efficiency generally decreases with increasing temperature, indicating a predominant physisorption mechanism, although some systems exhibit mixed physisorption–chemisorption behavior. Reported efficiencies vary widely depending on the plant extract type, acid concentration, and test temperature. For example, Equisetum arvense and Eucalyptus camaldulensis extracts showed high efficiencies at low temperatures with a marked decline above 60 °C, whereas mixture leaves extract from Fig and olive as well as Golpar leaves extract maintained or slightly increased efficiency at elevated temperatures, suggesting stronger adsorption. Other extracts, such as Date Palm leaves and seeds, Canarium Strictum leaves, and Pomegranate peels, followed Langmuir adsorption behavior, with optimal performance typically observed below 60 °C. These findings highlight the temperature-dependent nature of inhibitor performance and the importance of adsorption characteristics in defining their protective action. Although these studies have provided valuable insights, there is still room for improvement, particularly in exploring novel, sustainable inhibitors that maintain high efficiency across a wider temperature range and in simulating oilfield acidizing conditions more closely.

This study employs Allium porrum, Physalis peruviana juice, Physalis peruviana calyx (peels), and chicken eggshells as eco-friendly corrosion inhibitors due to their rich phytochemical composition, biological activity, and potential for waste valorization. Leek and Haranksh are rich in phenolic acids, flavonoids, carotenoids, and vitamins, offering strong antioxidant and antimicrobial properties. Haranksh peels, an abundant agricultural waste, contain high phenolic and flavonoid levels, while eggshells, composed mainly of CaCO₃ with a porous alkaline structure, are effective adsorbents with acid-neutralizing capacity^17–26^. As for corrosion studies, the aqueous extract of HP was reported to have anti-corrosive properties in steel/20% H_3_PO_4_ and steel/1 M HCl systems achieving IE of 80.0% using 0.4 g/L, and 91.4% using 1.0 g/L respectively^27,28^. Also, O. Sanni et al. used ES aqueous extract in steel/1 M HCl and stainless steel/0.5M H_2_SO_4_ systems achieving IE of 97.2% using 0.5 g/L, and 94.7% using 10.0 g/L respectively^26,29^. Leek and Haranksh juice were never used before for corrosion inhibition. In addition, there is no literature work reported for the usage of the four mentioned plants and agro-industrial wastes in the highly corrosive acidizing medium, 15% HCl.

These materials were selected based on:

1. Their constituents and bioactive components, which are rich in active centers, delocalized bonds, and aromatic rings, making them promising potential corrosion inhibitors.
2. Their availability and low cost in Egypt, with zero cost in the case of agro-industrial wastes.
3. Their green, sustainable, and economic benefits, converting waste into value-added, non-toxic materials for oilfield applications.

Their use aligns with sustainability goals, reduces environmental impact, and provides a practical, low-cost solution for corrosion mitigation under harsh acidizing conditions in the oil and gas industry.

This study aims to systematically evaluate the corrosion behavior of mild steel in 15% HCl in the presence of four novel, eco-friendly corrosion inhibitors-Allium porrum (Leek), Physalis peruviana (Haranksh) juice, Haranksh peels, and chicken eggshells-derived from natural and agro-industrial waste sources. The novelty of this work lies in the selection of underexplored inhibitors rich in bioactive compounds and active functional groups, combined with their low or zero cost, high availability in Egypt, and environmental sustainability. The temperature range of 30–60 °C was chosen to represent the operational conditions typically encountered in hydrochloric acid-based industrial processes, including oil well acidizing, pickling, and descaling, while also enabling the assessment of the inhibitors’ thermal stability and adsorption mechanisms under realistic, high-temperature environments. The inhibitors’ performance was comprehensively assessed using electrochemical impedance spectroscopy (EIS), potentiodynamic polarization, scanning electron microscopy (SEM) surface characterization, and adsorption isotherm modeling, providing mechanistic insights into their mode of action and potential for sustainable industrial application.

## Experimental

### Materials

Leek, Haranksh, and chicken eggshells were procured from a local market. Their collection and handling comply with all relevant institutional, national, and international guidelines and standards. High-purity analytical-grade reagents were used throughout the study, including 34% hydrochloric acid (HCl), absolute ethanol (99%), and double distilled water.

### Solution preparation

Green corrosion inhibitors were prepared from ethanolic extracts of Leek (LE), Haranksh Juice (HJE), Haranksh Peels (HPE), and Chicken Eggshells (ESE).

HJ was obtained by squeezing the Haranksh pulp and filtering out the seeds. The pulp is naturally soft and enclosed within a thin outer cover containing both juice and seeds. Squeezing the pulp is sufficient to rupture this thin cover, releasing the juice and seeds. The mixture is then filtered to remove the seeds, leaving only the juice for further use. For LE and HP extracts, the materials were washed thoroughly with tap water. In the case of ESE, the inner membrane of the eggshells was first removed prior to washing. All samples were then dried for one hour in the oven at 80 °C and ground to get a fine powder.

For each extract, 10 g of the powdered material was heated under reflux for one hour in 100 mL of absolute ethanol. The resulting solution was then filtered to eliminate impurities, and the final volume was adjusted to 100 mL with ethanol to prepare the stock solution. Then, 10 mL of this filtrate was pipetted in a clean and dry beaker of known weight (W_1_) by the usage a digital balance has ± 0.1 mg precision. The beaker was heated until ethanol solvent fully evaporated, and the remaining solid residue was left to cool and weighed (W_2_). The weight difference (W_2_ - W_1_) is the weight of extracted materials (grams) in 10 mL. Then using simple calculation gets the concentration in (g/L) of each stock solution.

All test solutions were prepared using 15% HCl (diluted from 34% HCl) and contained 10% ethanol. Appropriate volumes of each extract stock solution and double-distilled water were added to prepare the needed inhibitor concentration prior to each experiment.

### Electrochemical tests

Electrochemical Impedance Spectroscopy (EIS) and potentiodynamic polarization measurements were conducted using a “Parstat 2263” potentiostat/galvanostat, with data acquisition and analysis performed via Powersuite software. Platinum sheet was utilized as the counter electrode, while reference electrode composed of a saturated Ag/AgCl (KCl). The working electrode was fabricated from mild steel (ASTM A283/grade C) with the chemical composition (wt%): C 0.24; Mn 0.90; P 0.04; S 0.04; Si 0.35; Cu 0.20; balance Fe. The steel was cut and shaped into a cylinder, then mounted into a Teflon (Poly tetra floro ethylene PTFE) rod. Electrical contact was established via a copper wire, connected to one end of the steel cylinder and enclosed within a glass tube that functioned as both an insulator and a holder. Only one face of the steel (exposed area = 0.283 cm²) remained uncovered and was in contact with the test solution. The exposed surface was sealed to prevent leakage and mechanically abraded using emery papers of progressively finer grades, followed by rinsing with double-distilled water immediately prior to immersion. Prior to the electrochemical measurements, the working electrode was submerged in the test solution for 15 min to achieve stable open circuit potential (OCP).

Electrochemical Test Protocols

1. EIS: Conducted using a 10 mV of AC perturbation over a range of frequency 0.01-10⁴ Hz.
2. Potentiodynamic Polarization: Scans performed from ± 250 mV with respect to OCP at a rate of 30 mV/min.
3. All tests were conducted at 30, 40, 50, and 60 °C (± 0.1 °C), with each experiment conducted in triplicate to ensure reproducibility, maintaining an experimental error of less than 2%.

### Weight loss measurements

Steel coupons with dimensions (5 cm × 2 cm × 0.1 cm) were used in the weight loss study. The coupons were mechanically polished using emery papers to finer grades. Then, the polished steel specimens were weighted (W_1_) and immersed in 15% HCl blank alone, and after adding the four extracts for 20 h. Then, steel specimens were washed thoroughly with water, and ethanol, left to dry at room temperature, and then weighted again (W_2_) to calculate the inhibition efficiency.

### Characterization of steel surface by scanning electron microscopy (SEM)

The morphology of mild steel surface was inspected using Scanning Electron Microscopy (SEM) of the Model: JSM-200 IT, JEOL. Micrographs were captured at a 20 kV accelerating voltage and a 1.5kX magnification. Prior to imaging, the steel samples were mechanically abraded and then immersed for 1 h in 15% HCl solution, both without and with the addition of the four natural extracts (LE, HJE, HPE, and ESE). After exposure, the samples were gently rinsed and allowed to dry at room temperature before SEM observation.

## Results and discussion

### Corrosion behavior of steel in 15% HCl at 30 °C with plant extracts

**Fig. 1:**
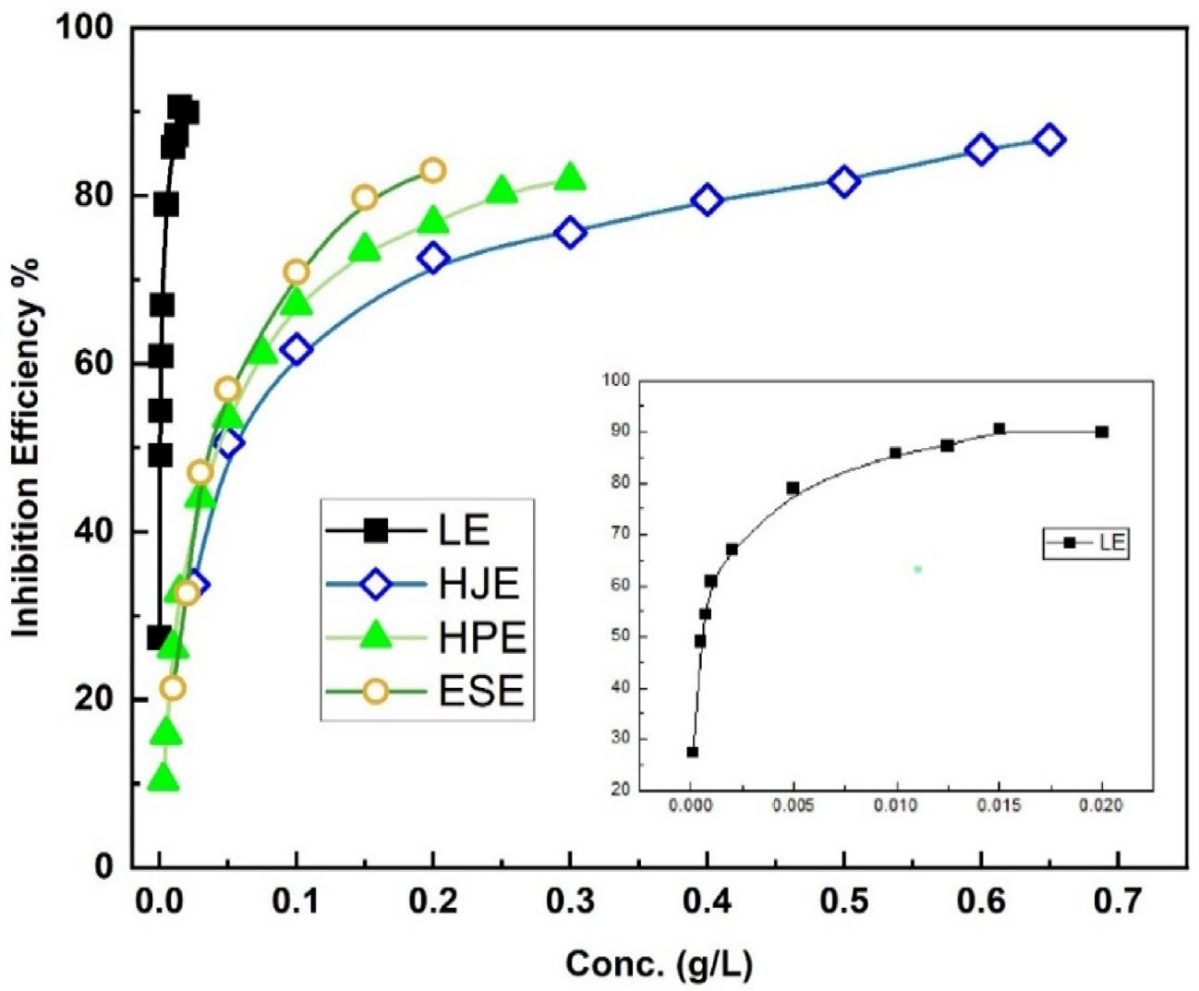
Corrosion inhibition efficiency variation of steel in 15% HCl in the presence of different concentrations of extracts at 30 °C.

The inhibition efficiency (%η) variation versus concentration for all tested green inhibitors—Leek Extract (LE), Haranksh Juice Extract (HJE), Haranksh Peels Extract (HPE), and Eggshell Extract (ESE)—in 15% HCl at 30 °C is illustrated in Fig. 1.

The %η–concentration profiles exhibit an initial sharp increase in inhibition efficiency, as a result to the protective monolayer film rapid formation on the steel surface. This is followed by a more gradual increase until reaching a plateau region at higher concentrations, indicating surface saturation by the inhibitor molecules. The saturation implies that surface coverage reached its maximum, and no further enhancement in efficiency is observed with increased inhibitor concentration.

### Temperature impact on the steel corrosion in 15% HCl with plant extracts

The effect of temperature on the behavior of steel corrosion was investigated at 40, 50, and 60 °C, in comparison to 30 °C, using 15% HCl solutions containing the selected optimal concentrations of each inhibitor: 0.010 g/L (LE), 0.600 g/L (HJE), 0.300 g/L (HPE), and 0.200 g/L (ESE). These concentrations were chosen as they correspond to the onset of the plateau region in the %η–concentration profiles (Fig. 1), representing the minimum concentrations required to achieve near-maximal inhibition efficiency.

**Fig. 2:**
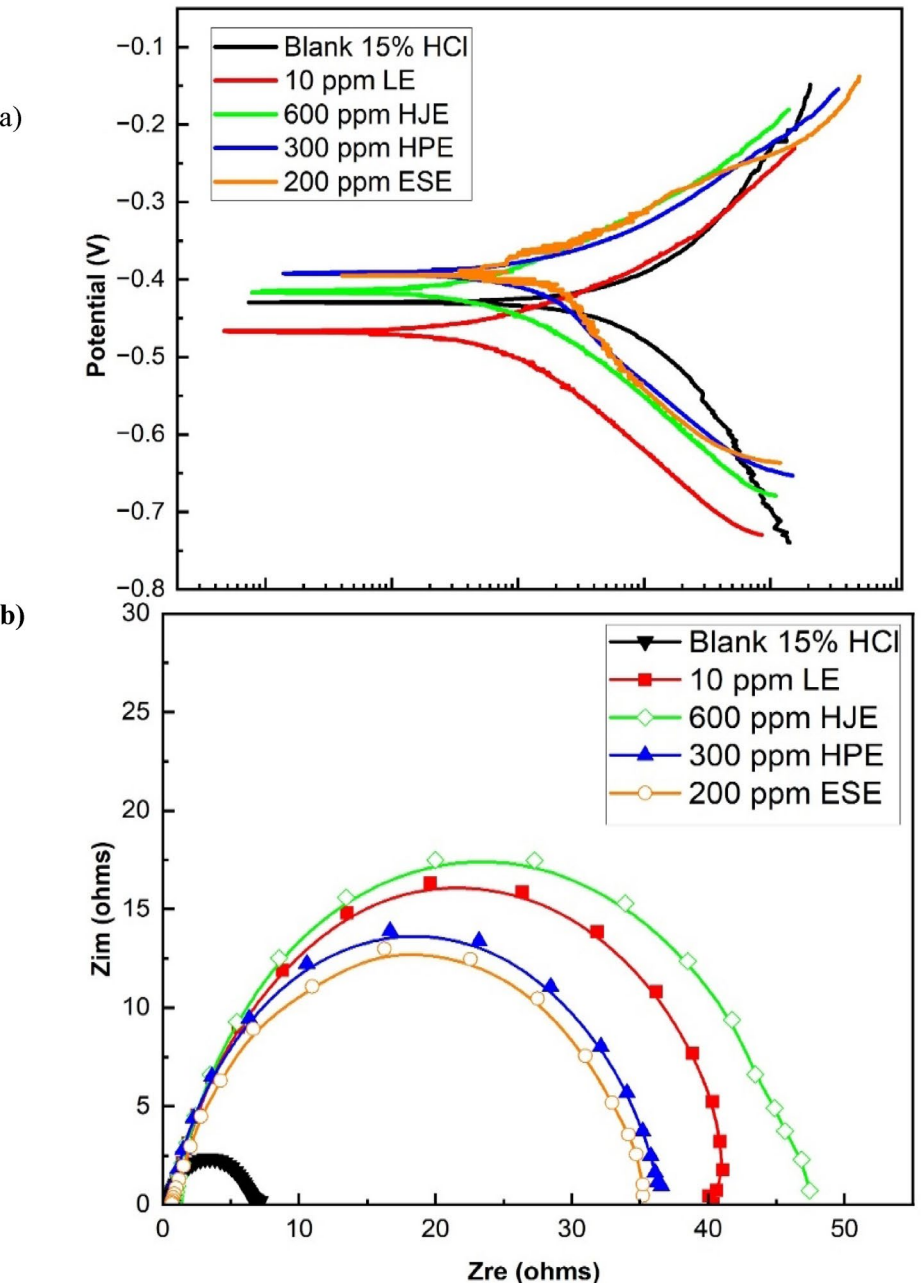
(**a**) Potentiodynamic polarization plots, (**b**) Nyquist impedance plots of steel in 15% HCl in the absence and after adding different concentrations of extracts at 30 °C.

Figure 2a and b present the Potentiodynamic polarization curves and Nyquist plots at for steel 30 °C, respectively, in 15% HCl solution, before and after adding the aforementioned extract concentrations. These plots provide insight into the corrosion kinetics and surface resistance characteristics conferred by the green inhibitors.

The corrosion current densities in the absence (i_0_), and presence (i) of inhibitor, attained from the polarization measurements, were used to calculate the inhibition efficiency, %η, using the following equation^2–5^. :

1 $$\% \eta \, = \, \left[ {\left( {i_{0} { - }i} \right) \, / \, i_{0} } \right] \, \times 100$$

**Fig. 3:**
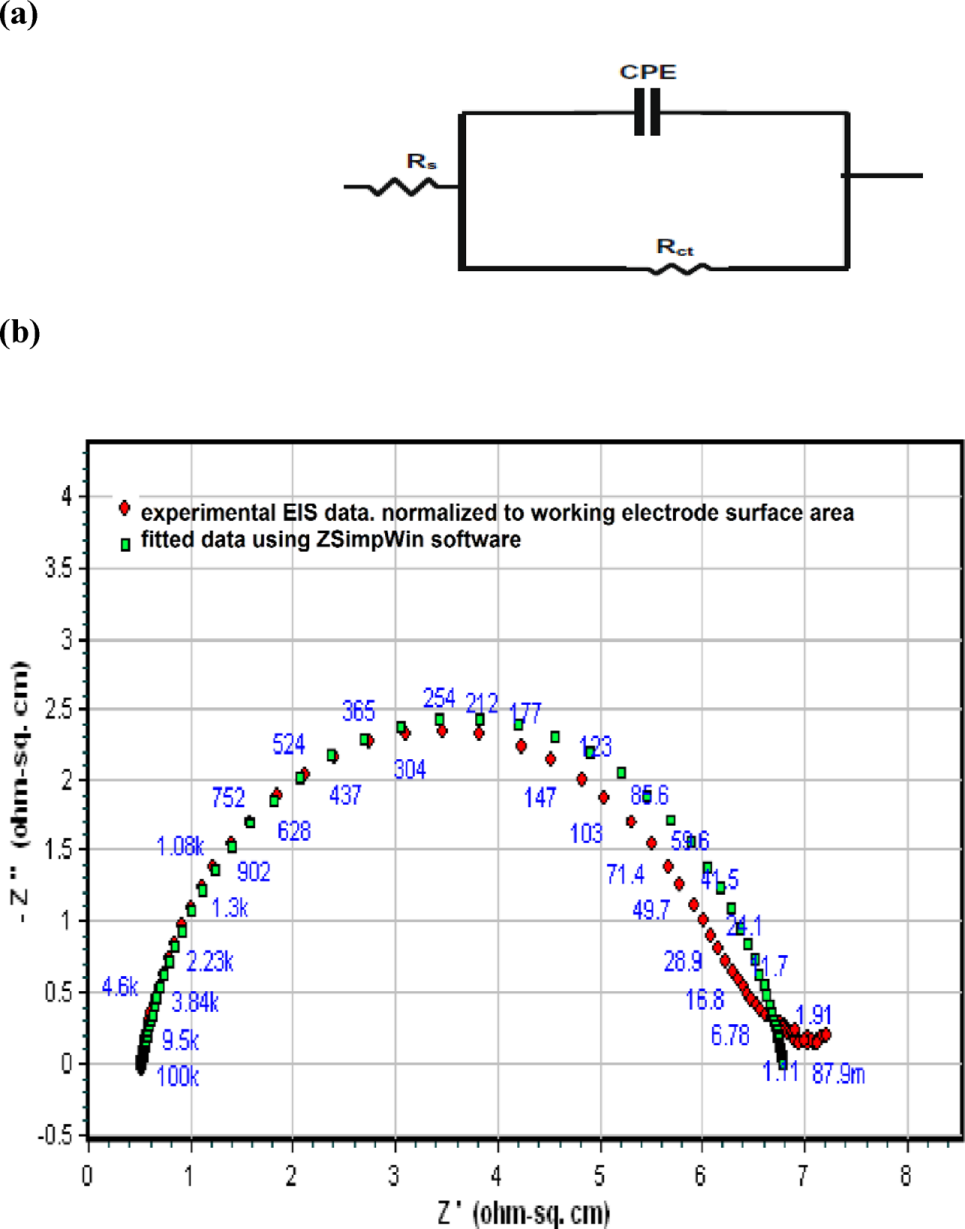
(**a**) The equivalent circuit model, (**b**) The representative figure of using the equivalent circuit to fit the experimental data for steel in 15% HCl.

The Nyquist impedance plots were analyzed by using the ZSimpWin program to fit experimental data to a simple equivalent circuit model R_s_ (Q_dl_R_ct_), that was used before in steel/acid interface^3–14^, as shown in Fig. 3a and b. The circuits include the solution resistance (R_s_) and constant phase element (CPE) that is placed in parallel to the charge transfer resistance element (R_ct_). The R_ct_ value is a measure of electron transfer across the surface and is inversely proportional to the corrosion rate. In a non-homogenous system, as in the case of a real steel/acid interface system, the capacitances were applied as CPE that is defined by the non-ideal-double layer capacitance (Q_dl_) and constant (n) that represents the phase shift, which is related to the system heterogeneity, where n values are less than 1, while Q_dl_, in (µs^n^/Ω.cm^2^), is numerically equal to CPE admittance (Y_o_). The CPE, that symbolizes the irregularity of electrode surface, makes the Nyquist semicircle to be greatly depressed^4,13,15^.

The impedance, Z, of CPE is presented by the Eqs^4,5,13^.

2 $$Z_{CPE} = \, Q_{dl}^{ - 1} \left( {i\omega } \right)^{ - n}$$

Where, the imaginary number i = (-1)^1/2^, ω is the angular frequency in rad.s^− 1^, ω = 2πf, and f is the AC frequency in Hz.

The steel electrode/solution interface forms a boundary with opposite charges which is known as double layer capacitance^17^. The ideal double layer capacitance (C_dl_) in (µF/cm^2^) could be calculated using the following equation^3,4,15^.

3 $$C_{dl} = \, \left( {Y_{0} \times \, R_{ct} } \right)^{1/n } / \, R_{ct}$$

The inhibition efficiency of mild steel by the weight loss measurements in acidic medium can be calculated by the Eqs^11,13^. :

4 $$\% \, \eta \, = \, 1 - \left( {\Delta W_{i} / \, \Delta W_{o} } \right)$$

where, ΔWi is the weight lost (g) in the presence of the extract while ΔW_o_ the weight lost (g) in the absence of the extract.

Table 1 shows the calculated parameters for electrochemical polarization and impedance, and weight loss of mild steel in 15% HCl solution, including the corrosion potential (E_corr_), cathodic (βc) and anodic (βa) tafel slopes, corrosion current density (i_corr_), inhibition efficiency (%η), charge transfer resistance (R_ct_), double layer capacitance (C_dl_), the weight loss in steel coupon ΔW, and the corrosion rate expressed in mmpy (mm/year).

**Table 1:** The electrochemical polarization, and impedance parameters, and weight loss measurements of steel in 15% HCl solution in the absence and presence of various concentrations of extracts at 30 °C.

Concentration (g/L)	PDP					EIS			Wt. loss	
	-E_corr_ (mV)	βa mV/decade	-β_C_ mV/decade	i_corr_ (µA cm^− 2^)	% η	*R*_ct_ (Ω cm^2^)	C_dl_ (µF/Cm^2^)	% η	ΔW (g)	% η
0.00	460	240	221	4055	–	6.27	1674	–	0.5920	–
0.010 g/L LE	504	135	146	577	85.8	40.47	1090	84.5	0.1142	80.7
0.600 g/L HJE	411	114	167	569	86.0	45.80	941	86.3	0.1103	81.4
0.300 g/L HPE	430	125	142	703	82.7	35.84	897	82.5	0.1239	79.1
0.200 g/L ESE	409	94	164	719	82.3	35.24	990	82.2	0.1259	78.7

The experimental data clearly demonstrate that the application of the tested extract concentrations reduces the corrosion current density (icorr), and increases the charge transfer resistance (Rct). This behavior indicates an enhanced surface coverage by the adsorbed molecules of the inhibitor, as reflected in the increased inhibition efficiency (η%)^27^.

The parallel nature of the tafel slopes suggests that the addition of the inhibitors does not change the fundamental corrosion mechanism and continues to be activation-controlled^14,27^. However, the four extracts, at the used concentrations, have a pronounced shift in Tafel slopes (βa and βc) with respect to blank, implying that the inhibitors influence both reactions of the hydrogen evolution and metal dissolution. This is further evidenced by the observed displacement of the polarization curves toward more noble potentials^27^.

Since the changes in E_corr_ (inhibitor) values after inhibitor addition are less than 85 mV relative to the blank solution, affecting both anodic and cathodic reactions. Therefore, the extracts can be categorized as mixed-type inhibitors^2,13^.

The Nyquist plots, in Fig. 2b, exhibit capacitive semicircles, which is in relation to electrical double layer and charge-transfer controlled corrosion process. The observed depression of these semicircles indicates the surface roughness and heterogeneity of the steel, as well the adsorption influence of the inhibitor. For the tested extracts, an increase in the semicircle diameter indicates a higher inhibition efficiency. Accordingly, HJE exhibits the highest inhibition efficiency, as evidenced by its largest semicircle diameter. Besides, all the plots exhibit similar shapes, suggesting that the extracts addition does not modify the mechanism, but only defeats the rate of charge transfer suggesting that adsorbed extract molecules effectively act as a diffusion barrier against aggressive ions^3–11,13^.

Furthermore, the trend of double-layer capacitance (C_dl_) values can be understood in the light of Helmholtz Eq^4^. :

5 $$C_{dl} = \, \varepsilon \, \varepsilon_{o} A \, / \, d$$

where ε is the dielectric constant of the medium, ε_o_ is the vacuum permittivity, A is the electrode surface area, and d is the thickness of the protective layer.

The observed decline in C_dl_ at these mentioned higher concentrations, as compared to blank, could result from either an expanded thickness of the electrical double layer or a reduced local dielectric constant. This effect likely occurs because organic compounds in the extracts displace water molecules at the interface of metal-solution, forming an adsorbed protective layer that impedes the corrosion process^3,4,14^.

The weight loss measurements at 30 °C in 15% HCl confirm the significant inhibitory effect of all four eco-friendly extracts on mild steel corrosion. The highest inhibition efficiency was recorded for HJE (81.4%), closely followed by LE (80.7%), indicating comparable performance among the extracts at their optimum concentrations. The relatively high efficiencies at such low concentrations highlight the strong adsorption capacity of the bioactive constituents present in the plant and waste-derived extracts. The weight loss results are in good agreement with the electrochemical data (EIS and potentiodynamic polarization), confirming that the corrosion inhibition mechanism is consistent across different measurement techniques. This agreement reinforces the reliability of the observed protective effects and validates the synergistic influence of the extracts’ phytochemical or mineral components in forming stable, adherent films on the steel surface.

### Application of adsorption isotherms

Adsorption isotherms are commonly employed to illustrate the interaction mechanisms between steel surfaces and plant-based inhibitors in acidic media such as 15% HCl solutions. The efficacy of corrosion inhibition is fundamentally governed by both the adsorption behavior of the inhibitor, as well as the surface physicochemical properties of the steel^13^.

In the present study, electrochemical polarization measurements conducted at 30 °C in 15% HCl, allowed for the quantitative determination of surface coverage (θ) by the plant extract molecules, calculated using the following relation^3,13,27^:

6 $$\theta \, = \% \eta /100$$

**Fig. 4:**
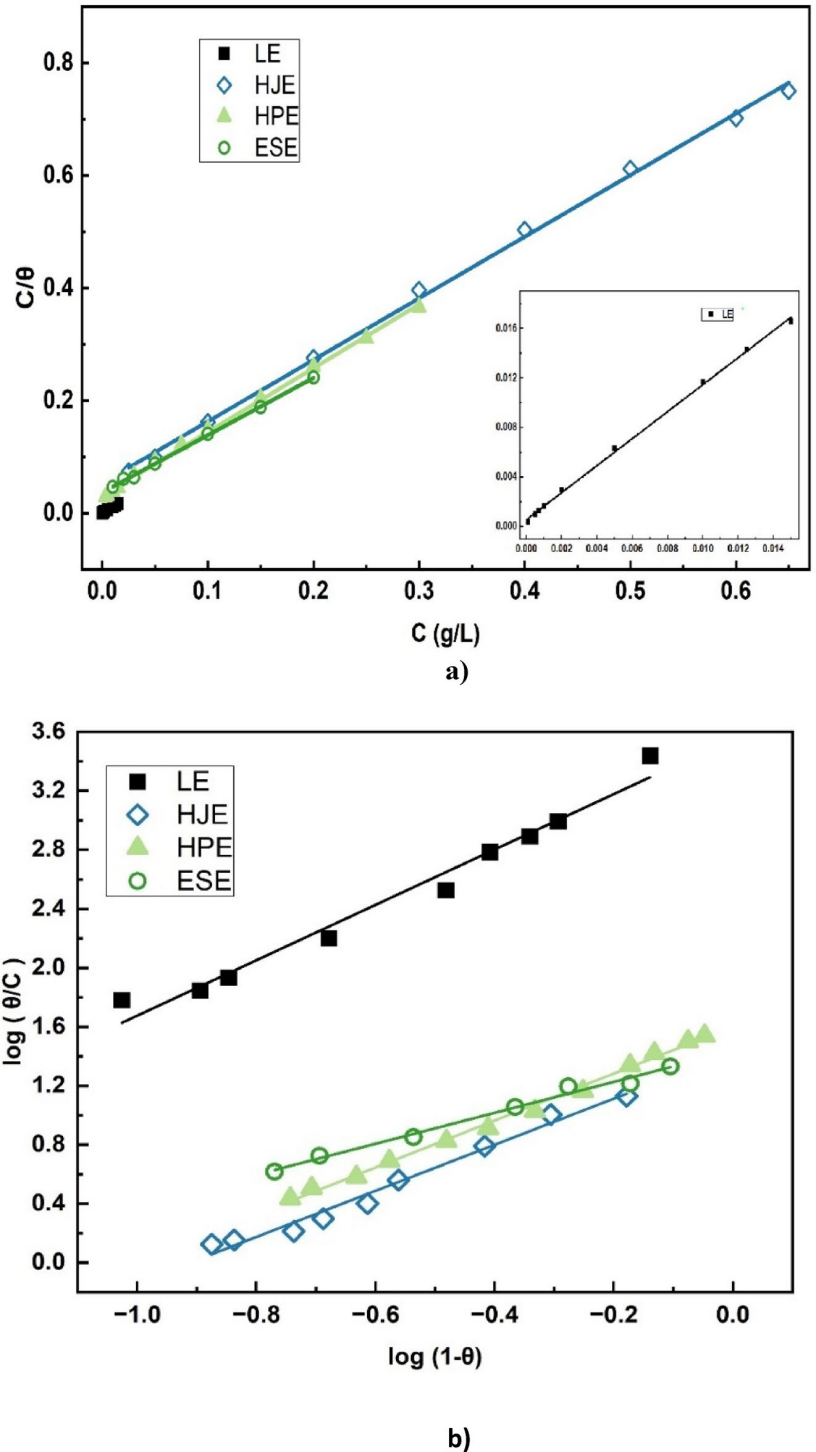

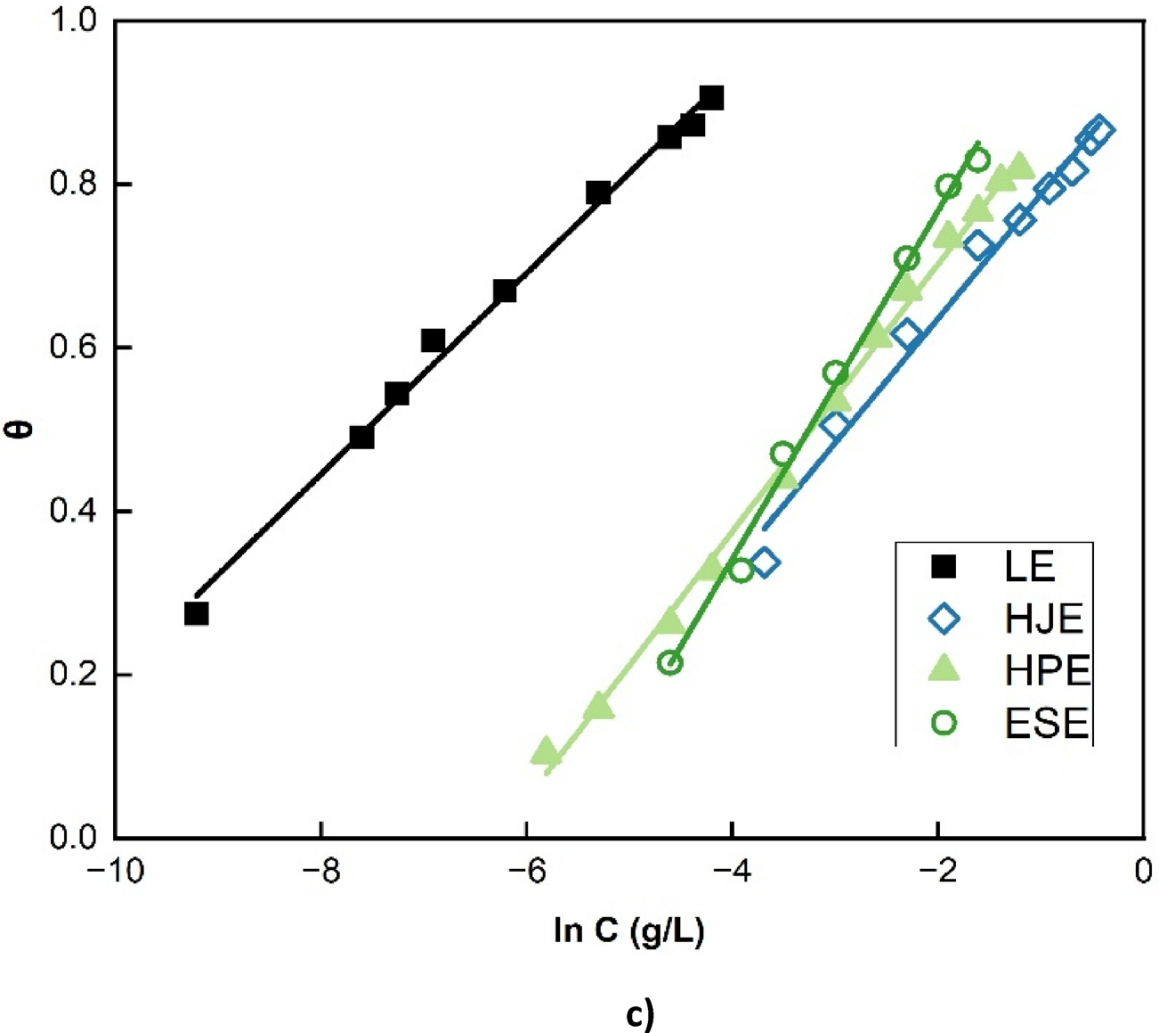
Fitting of (**a**) Langmuir, (**b**) Flory-Huggins, and (**c**)Temkin adsorption isotherms to the experimental polarization data of extracts.

These surface coverage values were subsequently applied to evaluate the adsorption behavior of the extracts by fitting them into several commonly used adsorption isotherm models—namely, Langmuir, Florry-Huggins, Temkin, and Frumkin adsorption isotherms^2,7,16,30^. The best fit model was determined based on the linear. correlation coefficient (R^2^) values obtained from the linearized forms of the isotherm Eqs. (6–9). As shown in Fig. 4, the Langmuir, Flory-Huggins, and Temkin adsorption isotherms exhibited strong linear correlations with the experimental data, suggesting that these models adequately describe the adsorption process of the extracts on the steel surface. Conversely, the Frumkin isotherm failed to produce a satisfactory linear fit, indicating that it does not suitably represent the adsorption behavior in this system.

7 $$Langmuir: C/\theta \, = \, 1/K + C$$

8 $$Flory - Huggins: \log \, (\theta /C) \, = \, \log \, K \, + \, x \, \log \left( {1 - \, \theta } \right)$$

9 $$Temkin: \theta \, = \, \left( {1/f} \right) \, \ln \, K \, + \, \left( {1/f} \right) \, \ln \, C$$

10 $$Frumkin: \ln \, [\theta \, / \, C\left( {1 - \, \theta } \right)] \, = \, \ln \, K \, + \, 2a\theta$$

The standard Gibbs free energy of adsorption can be deduced from the adsorption equilibrium constant (K_ads_) through the Eqs^2,7,8^. :

11 $$K_{ads} = \, 1/C \, . \, \exp ( - \Delta G_{ads} / \, RT)$$

where R denotes the universal gas constant, T represents the absolute temperature and the water concentration C (equals 1000 g/L because K_ads_ values are in g/L, instead of 55.5 in case of mol/L). Table 2 displays the fitting parameters from fitting various isotherms and the corresponding calculated ΔG◦_ads_.

Langmuir’s theory postulates that each inhibitor molecule occupy only one active site. The Temkin adsorption operates under the assumption of uniform binding energies distribution, with a linear adsorption heat decrease as surface coverage increases. This model accounts for interactions among the adsorbed molecules, reflecting surface heterogeneity and adsorbate–adsorbate repulsion. In contrast, the Flory-Huggins isotherm incorporates parameter x, which denotes the number of occupied active sites per inhibitor molecule. When x > 1, it suggests that a single inhibitor molecule displaces multiple water molecules, indicative of multilayer or non-ideal adsorption behavior^2^.

**Table 2:** Linear fitting parameters of extracts according to Langmuir, Flory-Huggins, Temkin and Frumkin adsorption isotherms in 15% HCl at 30℃.

Extract	Langmuir		Flory-Huggins				Temkin				Frumkin		
	K (g/L)	R^2^	K (g/L)	x	R^**2**^	ΔG_**ads**_ (kJ/mol)	f	a	K (g/L)	R^**2**^	a	K (g/L)	R^2^
Leek	1737.21	0.999	1903.97	1.88	0.987	-36.43	8.13	-4.07	111643.73	0.994	-1.54	8892.82	0.980
H. juice	18.61	0.998	17.04	1.56	0.991	-24.54	6.58	-3.29	482.59	0.983	-0.868	42.14	0.879
H. peels	32.05	0.998	25.07	1.59	0.998	-25.52	6.12	-3.06	541.10	0.997	-0.667	47.49	0.985
Egg shells	26.95	0.999	26.02	1.05	0.994	-25.61	4.70	-2.35	271.40	0.993	-0.055	27.69	0.125

Experimental analysis of the four plant extracts reveals x > 1 for LE, HJE, and HPE, signifying non-ideal adsorption behavior at the interface of metal-solution, likely due to the complex molecular structures and functional groups within these extracts. In contrast, x = 1 for ESE, implying ideal, one-to-one adsorption behavior on the steel surface in 15% HCl.

The Langmuir adsorption isotherm assumes monolayer adsorption of inhibitor molecules onto a homogeneous surface with no interaction between adsorbed species. The high correlation coefficients (R² = 0.998–0.999) obtained for all four extracts indicate that the experimental data fit the Langmuir model very well, confirming that adsorption occurs predominantly through a uniform monolayer formation on the mild steel surface.

The adsorption process spontaneity is affirmed by the high numerical values of the adsorption binding constants (K), all significantly exceeding greater than unity. These higher K values reflect strong interactions of the extract molecules with the steel surface, and correlate directly with enhanced inhibition efficiencies^2,27^.

The Temkin heterogeneity factor (f), and the Frumkin interaction factor (a), where, f = -2a, are used to characterize the nature of surface heterogeneity and molecular interactions within the adsorption layer. In this study, positive values of (f) were obtained for all extracts, indicating repulsive interactions between adsorbed molecules. Conversely, negative (f) values (not observed here) would have indicated attractive interactions^27,30^.

Moreover, the standard free energy of adsorption (ΔG◦_ads_) values were negative for all extracts, affirming a spontaneous process of adsorption. The calculated magnitudes of ΔG◦_ads_, which fall between − 20 and − 40 kJ/mol, suggest a mixed adsorption mechanism—encompassing both physisorption and chemisorption—for all studied systems^2,3,11^.

The inhibitive mechanism of the plant extracts is primarily as a result to their constituent molecules’ adsorption onto the mild steel surface. These constituent molecules form a protective barrier layer, which effectively minimizes acid-induced corrosion by reducing the active sites available for hydrogen ion attack. This film-forming action interrupts both anodic metal dissolution and cathodic hydrogen evolution reactions.

The protective action of organic corrosion inhibitors generally follows a substitution adsorption mechanism, whereby the dissolved inhibitor molecules (Inh.)_sol_ displace pre-adsorbed water molecules (H_2_O)_ads_ from the steel surface. This process can be represented by the following equilibrium reaction ]9,12,17[:

12 $$\left( {Inh.} \right)_{sol \, + } xH_{2} O_{ads} \leftrightarrow \left( {Inh.} \right)_{ads \, + } xH_{2} O_{sol}$$

13 $$Fe^{2 + } + \, \left( {Inh.} \right)_{ads} \leftrightarrow \left[ {Fe - \left( {Inh.} \right)} \right]_{ads}^{2 + }$$

Here, the parameter x represents the size ratio between inhibitor molecules and displaced water molecules, demonstrating that adsorption capacity depends on the inhibitor’s molecular size dimensions. The adsorption process is driven by the affinity of polar functional groups (e.g., –OH, –NH₂, –COOH, and aromatic rings) exist in the extract components, where their metal surface interactions can be electrostatic attraction, donor–acceptor interactions, or chemisorption, depending on the extract composition and surface conditions.

This indicates that upon adsorption, the inhibitor molecules efficiently reduce the exposed metal surface area of the metal, particularly at the cathodic sites, thereby decreasing the number of the active sites available for hydrogen evolution. Simultaneously, at the anodic sites, the metal dissolution is inhibited due to the formed protective adsorption film, leading to the suppression of iron oxidation processes^9^.

### Surface examination by SEM

**Fig. 5:**
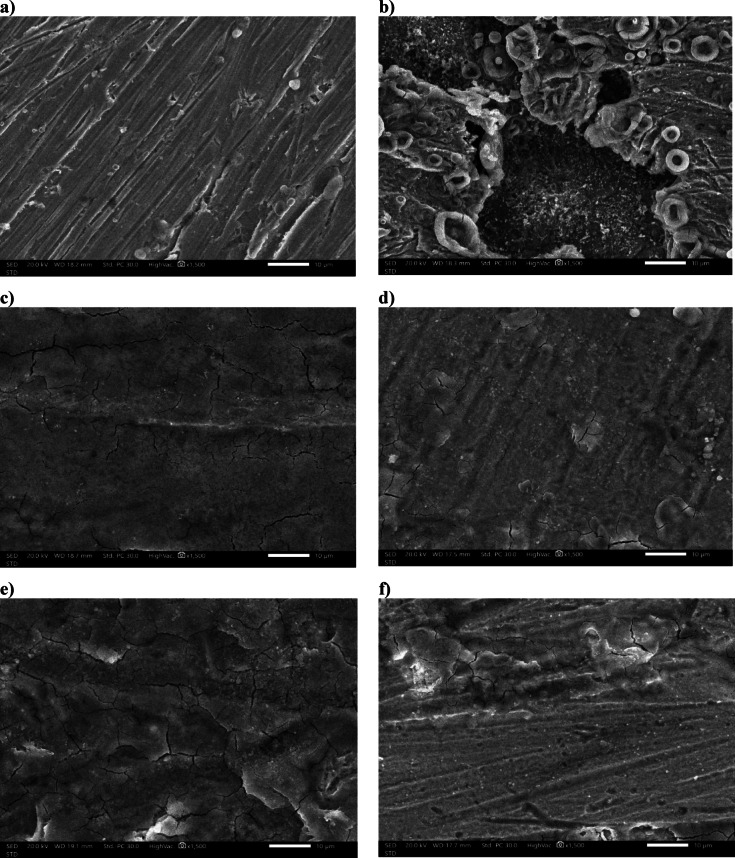
SEM micrograph of steel at 30 °C for: (**a**) polished, in (**b**) 15% HCl solution only: in the presence of (**c**) 0.010 g/L LE, (**d**) 0.600 g/L HJE, (**e**) 0.300 g/L HPE, and (**f**) 0.200 g/L ESE.

Figure 5 presents SEM micrographs of the polished mild steel surface at 30 °C under various conditions: (a) untreated, (b) exposed to 15% HCl without inhibitors, and (c–f) after exposure to the same acid medium containing LE, HJE, HPE, and ESE, respectively. As shown in Fig. 5a, the polished steel surface retains its characteristic abrasion marks, indicating minimal surface damage. In contrast, Fig. 5b reveals severe corrosion damage in the uninhibited 15% HCl solution, with a rough surface and evident shallow pits, confirming the aggressive nature of the acidic environment.

However, in the presence of the plant extracts (Fig. 5c–f), the steel surfaces exhibit significantly smoother morphologies with fewer visible defects. This suggests that the adsorbed bioactive constituents of LE, HJE, HPE and ESE onto the steel surface, causing the formation a protective layer that mitigated acid-induced corrosion. These morphological observations agree with the electrochemical impedance and polarization results, further confirming the inhibitory effectiveness of the studied extracts.

### Investigation of the temperature impact on the steel corrosion

Understanding the variation in inhibition efficiency with temperature is critical, as many industrial processes operate under elevated thermal conditions. Numerous previous studies have explored the temperature impact on the steel corrosion in hydrochloric acid media^1–8,10^. The present work investigates the influence of temperature (ranging from 30 to 60 °C) on the steel corrosion behavior both in the absence and presence of plant-based inhibitors.

#### Potentiodynamic polarization results

**Fig. 6:**
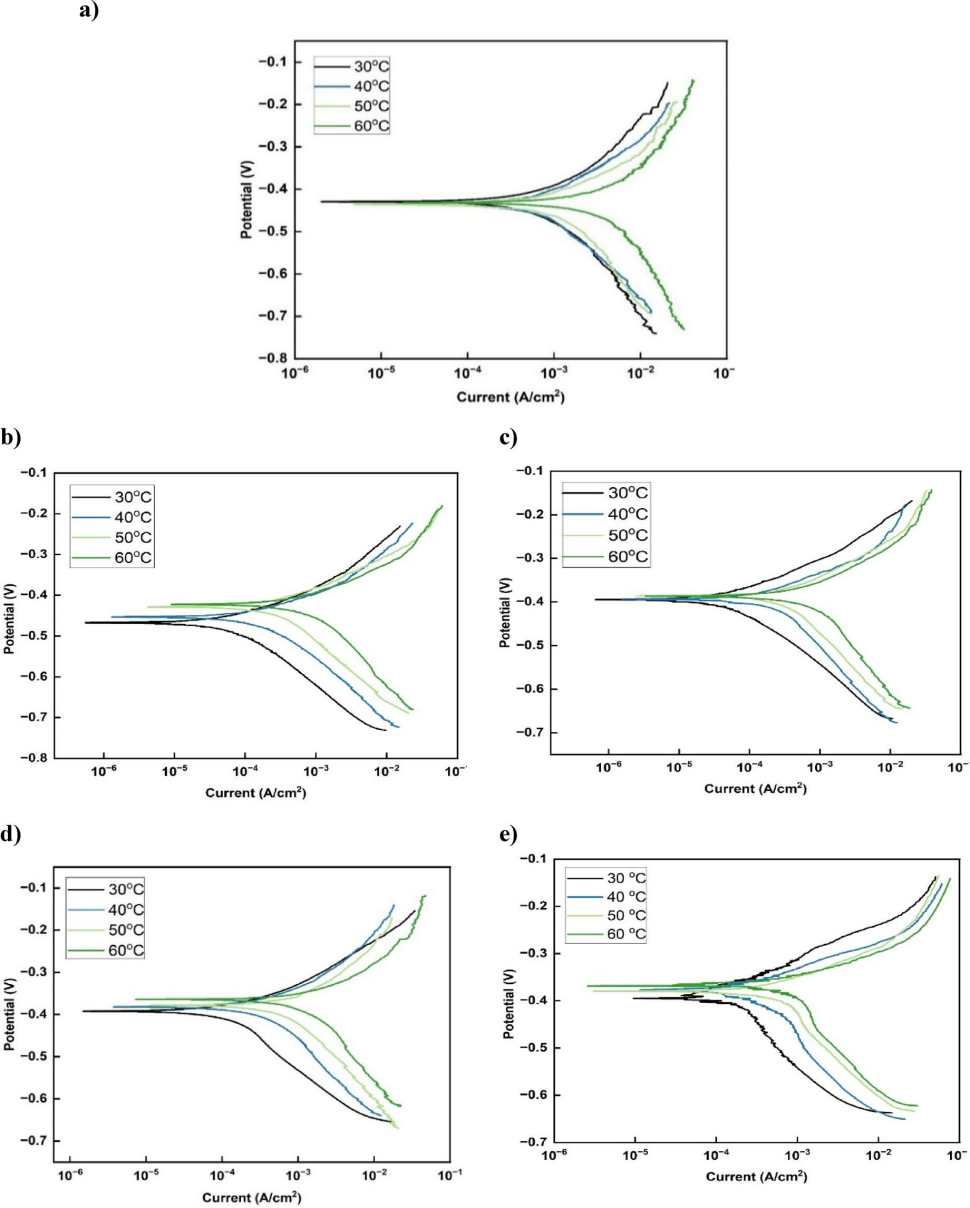
Potentiodynamic polarization plots of steel in 15% HCl solution at 30–60 °C: (**a**) in the absence and presence of (**b**) 0.010 g/L LE, (**c**) 0.600 g/L HJE, (**d**) 0.300 g/L HPE, and (**e**) 0.200 g/L ESE.

The potentiodynamic polarization curves presented in Fig. 6 demonstrate that increasing the temperature intensifies both the reaction of iron dissolution at the anode and hydrogen evolution at the cathode, even when inhibitors are present. This results in elevated corrosion current density (i_corr_) values, signifying accelerated corrosion rates at higher temperatures. The observed behavior is attributed to a probable thermal desorption of inhibitor molecules from the steel surface, thereby reducing the protective efficacy of the adsorbed film^8,27^.

#### Electrochemical impedance spectroscopy results

**Fig. 7:**
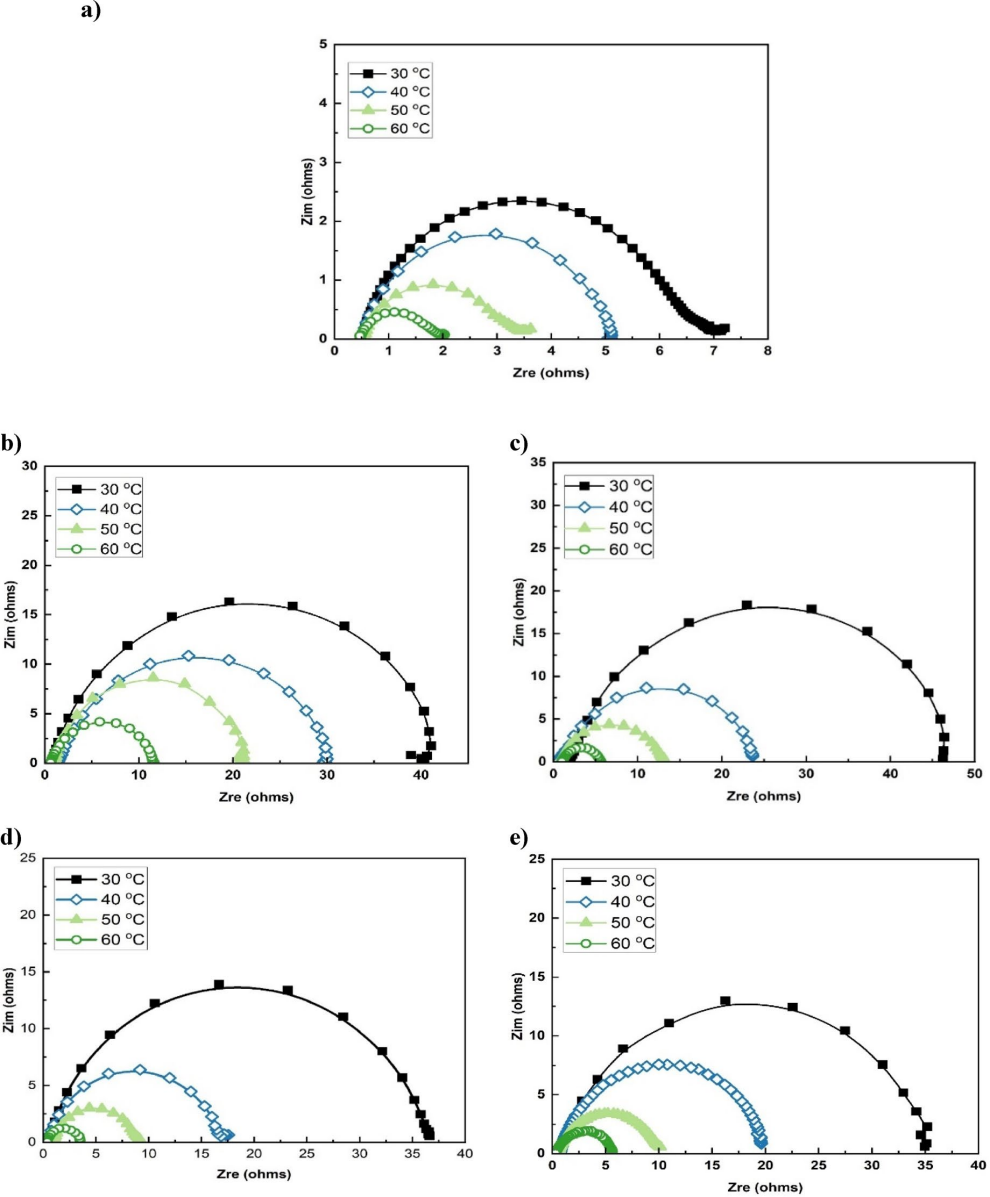
Nyquist impedance plots of steel in 15% HCl solution at 30–60 °C: (**a**) in the absence and presence of (**b**) 0.010 g/L LE, (**c**) 0.600 g/L HJE, (**d**) 0.300 g/L HPE, and (**e**) 0.200 g/L ESE.

Figure 7 displays the EIS Nyquist plots of mild steel in 15% HCl across a temperature range 30–60 °C, both in the absence and presence of the optimal concentrations of LE (0.010 g/L), HJE (0.600 g/L), HPE (0.300 g/L), and ESE (0.200 g/L). In all cases, the plots exhibit depressed semicircles, characteristic of a charge transfer-controlled corrosion process. The shape and nature of the plots indicate that in the presence of the plant extracts, the fundamental corrosion mechanism does not alter. However, with increasing temperature, the semicircle diameter progressively decreases, indicating a reduction in charge transfer resistance (R_ct_) and, consequently, raising of corrosion rate. This behavior suggests partial desorption of inhibitor molecules at higher temperatures, from the steel surface, thereby diminishing their protective effect^13,27^.

**Fig. 8:**
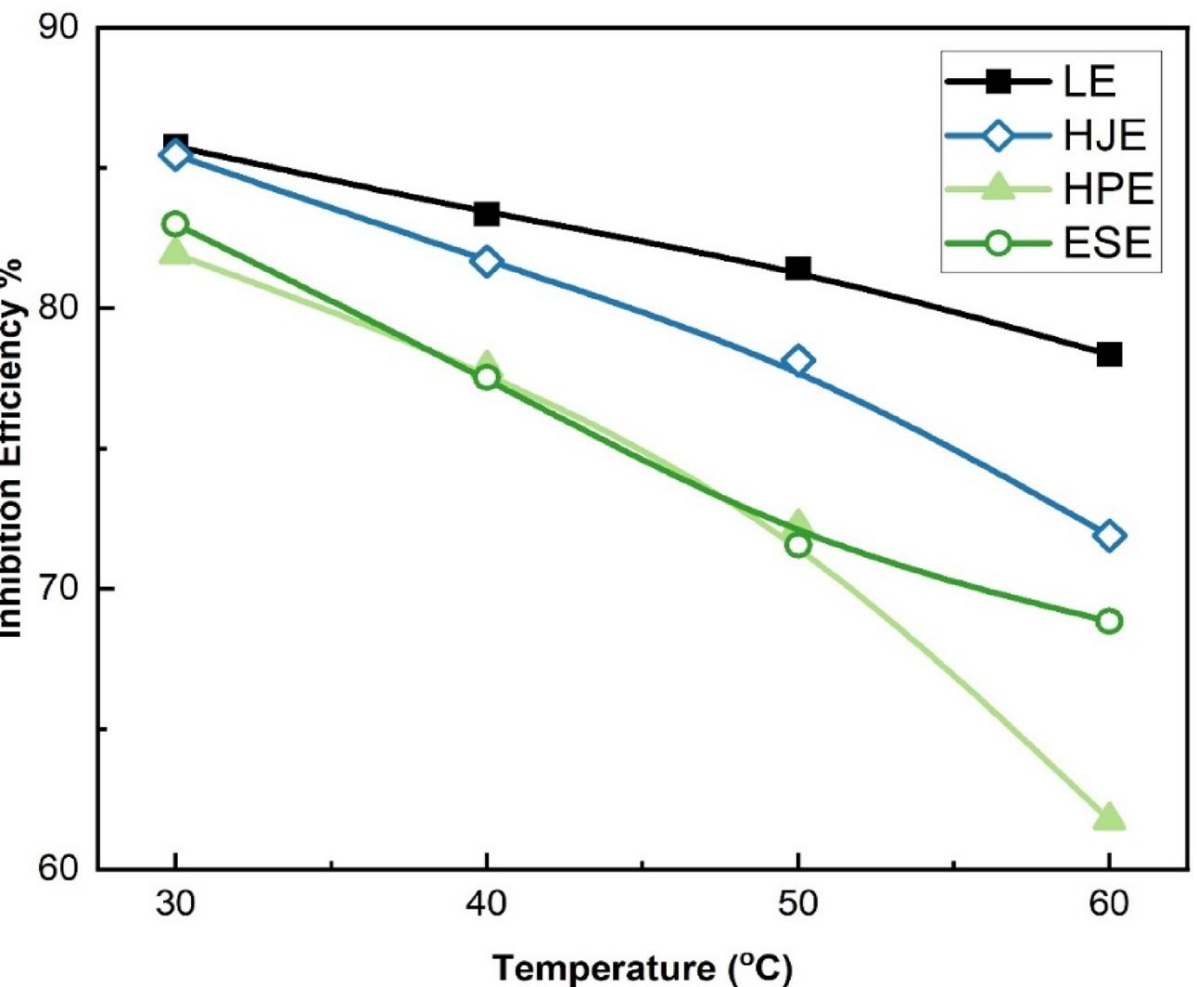
The relation between the inhibition efficiency and temperature for LE, HJE, HPE, and ESE on steel in 15% HCl solutions.

Figure 8 illustrates the change of inhibition efficiency, calculated from polarization measurements for the four plant extracts, as a function of temperature in 15% HCl solution. Across the studied temperature range (30–60 °C), the inhibition efficiency follows the order: LE > HJE > ESE > HPE, suggesting that the protective film formed by LE is the most stable and resilient to thermal degradation.

**Fig. 9:**
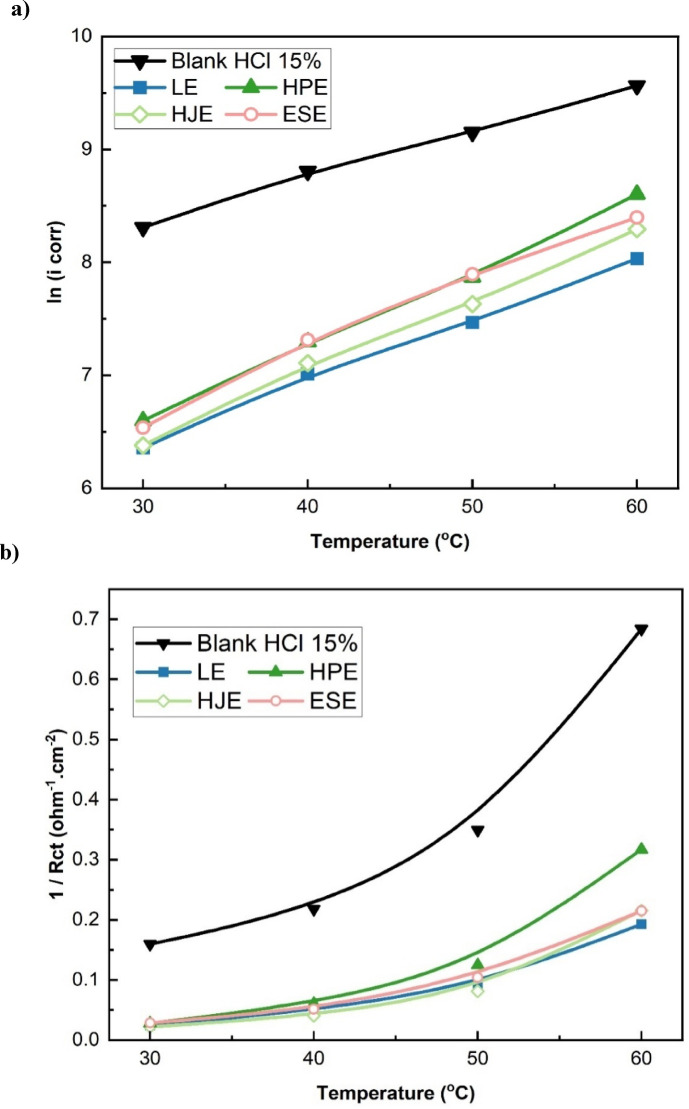
The variation of the corrosion rate of steel with temperature for LE, HJE, HPE, and ESE on steel in 15% HCl solutions in terms of change of (**a**) ln (i corr) and (**b**) (1/Rct).

This trend implies a slower breakdown of the adsorbed inhibitor layer in a similar order as temperature increases. The observed decline in inhibition efficiency at elevated temperatures is primarily attributed to the accelerated corrosion kinetics and the potential inhibitor molecules’ desorption from the steel surface^12^. As further illustrated in Fig. 9, this this behavior is corroborated by: (a) a marked increase in corrosion current density (i_corr_), and (b) a decrease in charge transfer resistance, reflected by an increase in 1/Rct. These results confirm the temperature-sensitivity of the inhibition process and highlight the need to consider thermal stability when selecting corrosion inhibitors for high-temperature applications.

**Fig. 10:**
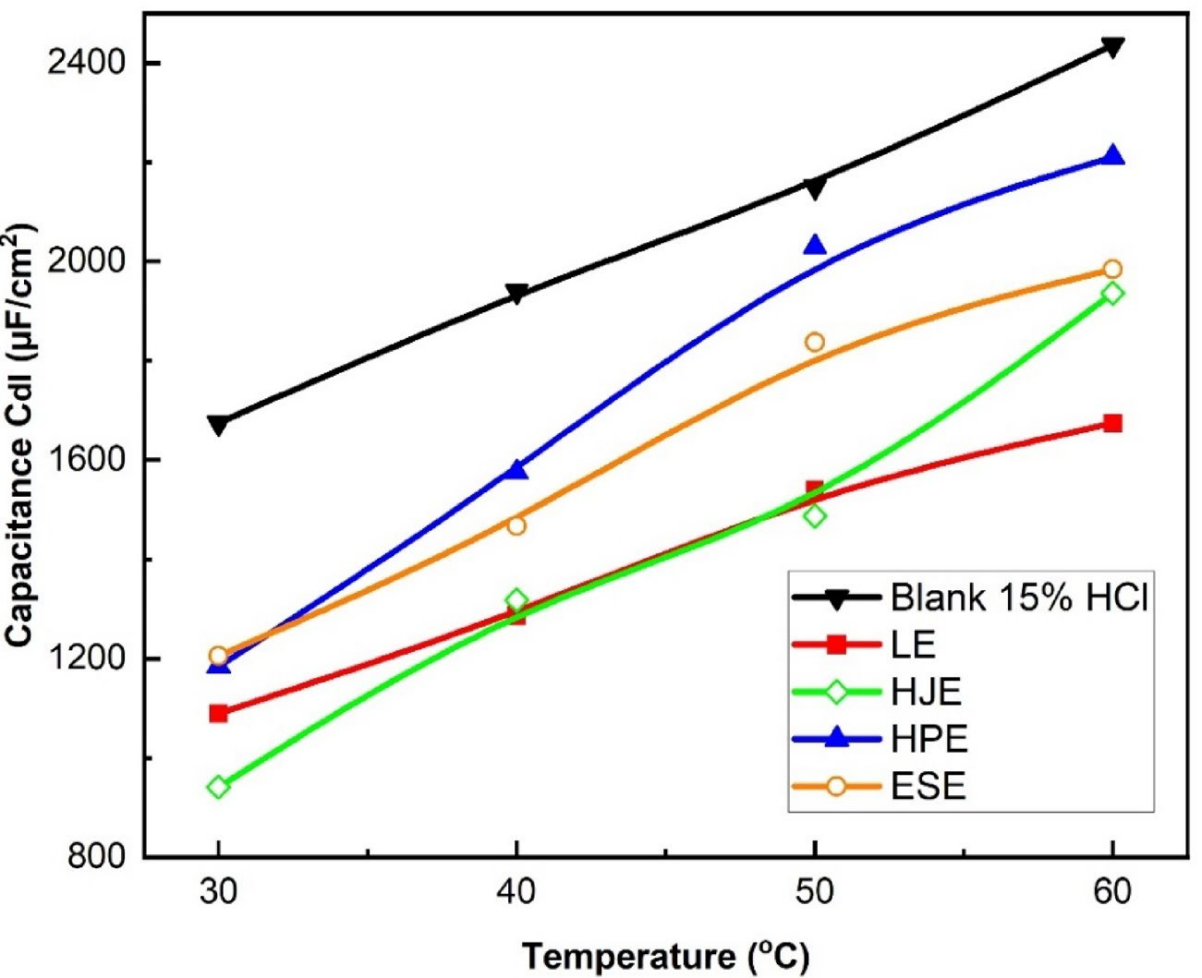
The change in the electrode double-layer capacitance with temperature for LE, HJE, HPE, and ESE on steel in 15% HCl solutions.

As the temperature rises from 30 to 60 °C, the double-layer capacitance (Cdl) in the acidic medium rises significantly. In the presence of the four plant extracts,, Cdl also shows a notable increase, which can be attributed to the desorption, thermal decomposition, or molecular rearrangement of inhibitor species adsorbed on the steel surface. These changes reduce the compactness and stability of the protective layer, leading to a lower surface coverage and hence a decline in inhibition efficiency^2,5^, as shown in Fig. 10. This increase in Cdl at elevated temperatures is consistent with the enhanced kinetic energy of the system, which disrupts inhibitor–metal interactions. As a result, the adsorption of extract molecules becomes less favorable, and desorption becomes dominant, weakening the barrier effect provided by the organic inhibitors^3^.

With rising temperature, the inhibition efficiency declines observably, suggesting that desorption of the protective species from the steel surface becomes thermodynamically favored over adsorption. This behavior is characteristic of physisorbed inhibitors, where elevated temperatures weaken the weak intermolecular forces binding the inhibitor to the surface. Two additional factors may intensify this temperature-induced efficiency loss:

1. Enhanced interfacial turbulence caused by the accelerated hydrogen evolution reaction, which mechanically disrupts the stability and continuity of the inhibitor film.
2. Increased surface roughness resulting from more aggressive corrosion at higher temperatures, which can reduce the number of effective adsorption sites and impair the ability of inhibitor molecules to maintain a coherent protective layer^9,27^.

Together, these effects contribute to the degradation of inhibitor performance at elevated temperatures, highlighting the importance of thermodynamic and kinetic evaluations in inhibitor design.

#### Determination of the activation parameters

The activation parameters for steel corrosion for steel in 15% HCl were determined in the absence and presence of 0.010 g/L LE, 0.600 g/L HJ, 0.300 g/L HP, and 0.200 g/L ES. by applying the Arrhenius and transition state Eqs^3,6,13^. In these models:

14 $$k \, = \, A \, e^{( - Ea/RT)}$$

15 $$k = RT/Nh\,e^{{\left( {\Delta S * /R} \right)}} e^{{\left( { - \Delta H * /RT} \right)}}$$

Where A represents the pre-exponential factor, R is the universal gas constant, T indicates the absolute temperature, N stands for the Avogadro`s number, h is the Planck’s constant, while, E_a_, ΔS^*^ and ΔH^*^ corresponds to the apparent activation energy entropy, and enthalpy.

**Fig. 11:**
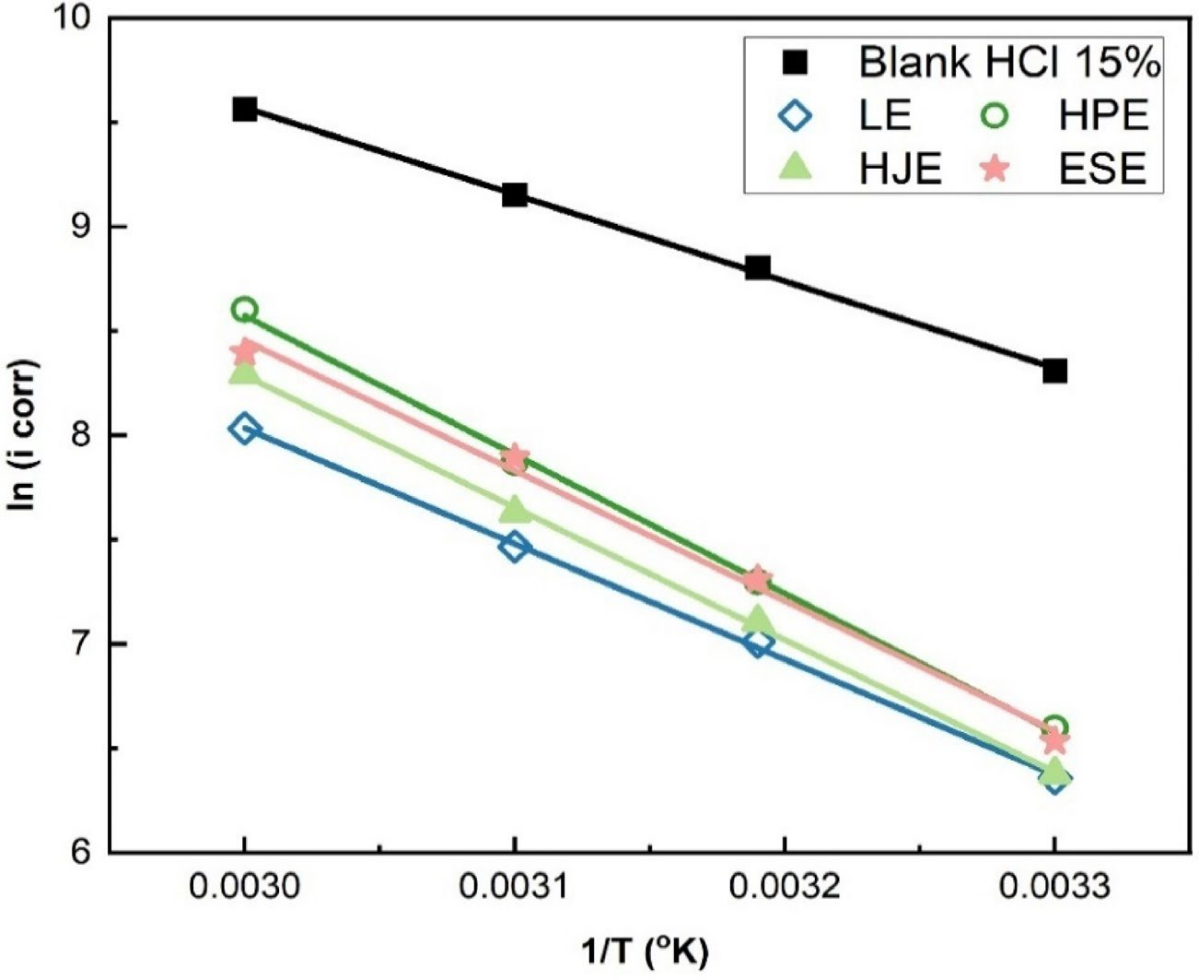
Linear fitting of ln(i_corr_) vs. (1/T) for steel in 15% HCl solution in the absence and presence of LE, HJ, HP, and ES extracts.

**Fig. 12:**
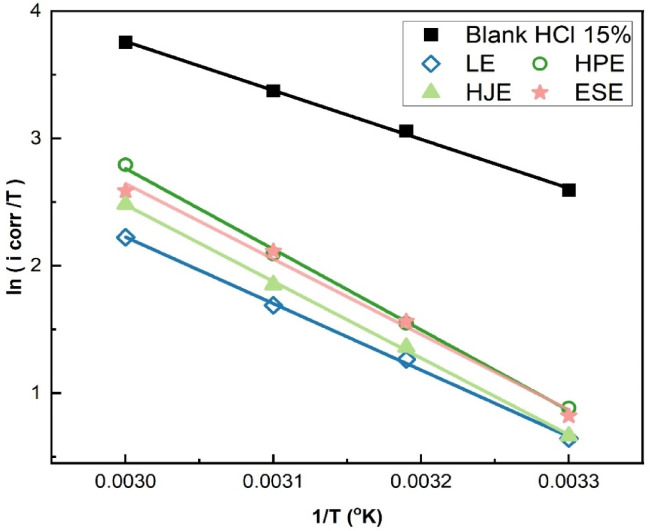
Linear fitting of ln(i_corr_ /T) vs. (1/T) for steel in 15% HCl solution in the absence and presence of LE, HJ, HP, and ES extracts.

The corrosion rates (k) were calculated from the corrosion current densities (i_eorr_) derived from potentiodynamic polarization at temperatures from 30 to 60 °C, both with and without the plant-based inhibitors. Figures 11 and 12 show the linear square fitting of “ln(i_corr_)” and “ln(i_corr_/T)” data vs. (1/T), in the absence and presence of extracts.

These plots confirm two significant observations:

1. A progressive reduction in adsorption capacity and inhibition efficiency with increasing temperature.
2. A corresponding increase in corrosion rate, indicative of thermal desorption of inhibitor molecules.

The calculated activation parameters are compiled in Table 3, alongside their respective correlation coefficients (R²), offering deeper thermodynamic understanding of the corrosion inhibition mechanism.

**Table 3:** Thermodynamic activation parameters for LE, HJ, HP, and ES extract.

Extract	A (µA cm^− 2^)	E_a_ (kJ mol^− 1^)	*R* ^2^	ΔH* (kJ mol^− 1^)	ΔS* (J mol^− 1^ K^− 1^)	*R* ^2^
–	3.69 × 10^9^	34.57	0.999	31.93	-70.46	0.999
0.010 g/L LE	4.99 × 10^10^	46.07	0.999	43.43	-48.75	0.999
0.600 g/L HJ	6.92 × 10^11^	52.67	0.999	50.03	-26.85	0.999
0.300 g/L HP	2.39 × 10^12^	55.31	0.999	52.67	-16.55	0.999
0.200 g/L ES	6.30 × 10^11^	51.86	0.994	49.22	-27.87	0.994

The activation energy (E_a_) values reported by J. Haque et al.^10^ for steel corrosion in 15% HCl align closely with the values measured in this study. Notably, both E_a_ and the pre-exponential factor (A) were significantly elevated upon the addition of plant extracts to the acidic solution. This increase in activation energy suggests that the extracts’ active compounds are adsorbed efficiently on the steel surface, which raises the energy barrier for corrosion. The inhibition occurs via two primary mechanisms:

1. Formation of a thicker protective double layer.
2. Modification of the steel/solution interface.

These observations underscore the extracts’ strong inhibitory capabilities, consistent with previous findings^3,7^.

The positive values of ΔH^*^ (activation enthalpy) indicate that the formation of the activated complex is an endothermic process^3,6,7^. The increase in ΔH^*^ in the presence of extracts likely results from the adsorption process, which elevates the energy required for the corrosion reaction^14^. Conversely, the negative values of ΔS^*^ (activation entropy) suggest an associative mechanism, whereby the transition from reactants to the activated complex is accompanied by a decrease in system disorder. This reflects the enhanced molecular organization induced by inhibitor adsorption onto the metal surface^8,9^.

The consistent observation that E_a_ > ΔH⁺ implies a corrosion mechanism involving gas evolution, specifically hydrogen generation, which results in an overall reduction in volume. The thermodynamic relationship between E_a_ and ΔH^*^ is described by the Eqs^4,13^. :

16 $$\Delta H* \, = E_{a} - \, \Delta n \, RT$$

where *R* is the universal gas constant, *T* is the absolute temperature, and *Δn* represents the difference in the number of moles between products and reactants. For the steel-HCl system with and without inhibitors, the experimentally derived ΔnRT ≈ 2.62 kJ/mol, which closely matches the theoretical value of 2.63 kJ/mol at 30 °C, supporting the assumption of a unimolecular corrosion reaction^4,13^.

## Conclusions

This study evaluated the performance of corrosion inhibition of four plant-based ethanol extracts-LE, HJE, ESE, and HPE- on mild steel in 15% HCl solution, simulating the acidic conditions encountered in oil well operations, over a temperature range of 30–60 °C.

Key findings include:

1. All four extracts demonstrated significant corrosion inhibition across the 30–60 °C range, for mild steel in 15% HCl, nonetheless, notable protection (61.8–85.8%) was maintained even at 60 °C, using relatively low concentrations (0.010–0.600 g/L). Thermodynamic activation parameters were calculated and interpreted.
2. Polarization curves confirmed that the extracts act as mixed-type inhibitors, while EIS and weight loss tests indicated effective surface coverage and formation of a protective film.
3. Adsorption isotherms fitting confirmed a spontaneous adsorption involving both physisorption and chemisorption, which is multi-site and non-ideal for all extracts except ESE.
4. SEM analysis revealed smoother steel surfaces in the presence of the extracts, supporting the formation of an adsorbed protective film composed of extract constituents.
5. Overall, the four extracts proved to be highly effective, sustainable corrosion inhibitors for mild steel in acidic environments typical of oil well acidizing operations. Their use offers additional environmental, economic, and waste-management benefits, supporting their potential application in the oil and gas industry.

## Data Availability

The datasets generated during and/or analyzed during the current study are available from the corresponding author on reasonable request.
